# Fonsecazyma yulaniae sp. nov., a yeast species isolated from flowers

**DOI:** 10.1099/ijsem.0.006830

**Published:** 2025-06-26

**Authors:** You-Jun Liao, Xuan Zhang, Zi-Xuan Liu, Rui Wang, Ya-Jing Yu, Lu Xue, Ai-Hua Li

**Affiliations:** 1 School of Biotechnology and Food Science, Tianjin University of Commerce, Tianjin, PR China; 2 China General Microbiological Culture Collection Center (CGMCC), Institute of Microbiology, Chinese Academy of Sciences, Beijing 100101, PR China; 3 Tianjin Institute of Industrial Biotechnology, Chinese Academy of Sciences, Tianjin 300308, PR China

**Keywords:** flower, *Fonsecazyma*, novel species, phylogeny

## Abstract

Two basidiomycete yeast strains, designated as 21S12 and 12S11, were isolated from the flowers of *Yulania denudata* collected from the Beijing Olympic Forest Park, PR China. Molecular phylogenetic analyses based on the D1/D2 domains of the large subunit rRNA gene and the internal transcribed spacer (ITS) region revealed that these strains represent a novel species within the genus *Fonsecazyma*. The new species, *Fonsecazyma yulaniae* sp. nov., is most closely related to *Fonsecazyma mujuensis* CBS 10308^T^, with sequence divergences of 4.3% (28 substitutions and 2 indels) in the D1/D2 domain and 8.4% (28 substitutions and 10 indels) in the ITS region. Phenotypically, *F. yulaniae* sp. nov. differs from *F. mujuensis* CBS 10308^T^ in its ability to assimilate inulin and creatinine, as well as its inability to assimilate lactose and erythritol. Additionally, *F. yulaniae* sp. nov. can grow in a vitamin-free medium and in a medium supplemented with 50% glucose, conditions under which *F. mujuensis* cannot grow. The holotype of *F. yulaniae* sp. nov. is CGMCC2.5852^T^, and its taxonomic description has been registered in Fungal Names (FN572294) and in Mycobank (MBT10026390).

## Introduction

The genus *Fonsecazyma* was established in 2015 by Liu *et al*. [1] to accommodate a distinct clade within the family *Bulleraceae* (order *Tremellales*, class *Tremellomycetes*). This clade, which exhibited strong phylogenetic support, initially included *Cryptococcus mujuensis*, *Cryptococcus tronadorensis* and *Kwoniella betulae*. Due to significant genetic divergence from other members of the genus *Cryptococcus* and the lack of close relatives for *K. betulae* within *Kwoniella*, these species were reclassified under the newly proposed genus *Fonsecazyma* [1]. However, in 2020, *Fonsecazyma betulae* and *Fonsecazyma tronadorensis* were transferred to the newly established genus *Teunia*, where they were renamed *Teunia betulae* and *Teunia tronadorensis*, respectively [2]. In 2024, Ghobadi *et al*. isolated a novel strain IRAN 18507F from Persian oak (*Quercus brantii*) branch in Iran and proposed it as a novel species of *Fonsecazyma*, *Fonsecazyma quercina* [3]. As a result, *Fonsecazyma* currently comprises two recognized species, *Fonsecazyma mujuensis* and *F. quercina*, while the newly published species *F. quercina* was not clustered together with the type species of *Fonsecazyma*, *F. mujuensis,* but clustered with some strains that seemed to be members of the genus *Teunia*.

Flowers are well-known habitats for yeasts [4], and numerous yeast strains have been isolated from floral sources, including *Vishniacozyma floricola* [5], *Wickerhamiella lachancei* [6], *Hannaella floricola* [7] and *Starmerella kisarazuensis* [8]. During a survey of yeast diversity associated with flowers, two basidiomycetous yeast strains, designated as 21S12 and 12S11, were isolated from the flowers of *Yulania denudata* in the Olympic Forest Park, Beijing, PR China. Phylogenetic analysis based on the D1/D2 domains of the large subunit (LSU) rRNA gene and the internal transcribed spacer (ITS) region revealed that these strains represent a novel species within the *Fonsecazyma* clade. Based on their distinct morphological, physiological and genetic characteristics, we propose it as a new species, with the name *Fonsecazyma yulaniae* sp. nov.

## Methods

### Sampling and yeast isolation

Flowers of *Y. denudata* were collected from the Olympic Forest Park in Beijing, PR China (40.02° N 116.40° E). The floral samples were collected with sterile tools and placed in sterile plastic bags, stored at 4 °C and transported to the laboratory for immediate processing. Approximately 5 g of floral stamen and petal material was suspended in 20 ml of sterile water and vigorously shaken to release microbial cells. Serial dilutions of the suspension were prepared, and 100 µl of each dilution was spread onto yeast extract-malt extract (YM) agar plates (containing 1.0% yeast extract, 2.0% malt extract, 0.4% glucose and 2.0% agar) supplemented with 100 mg l^−1^ chloramphenicol to inhibit bacterial growth. Three replicate plates were prepared for each dilution and incubated at 25 °C for 7 days. Yeast colonies that emerged were purified through repeated streaking on YM agar plates. Pure isolates were preserved by freeze-drying and stored in liquid nitrogen at the China General Microbiological Culture Collection Center (CGMCC).

### Phenotypic characterization

The morphological, physiological and biochemical characteristics of the strains were evaluated using standard methods as described by Kurtzman *et al*. [9]. Carbon assimilation tests were performed in a liquid medium of yeast nitrogen base (Difco, 291940), while nitrogen assimilation tests were conducted in a liquid medium of yeast carbon base (Difco, 239110), using starved inocula for the latter [10]. Fermentation tests were carried out using Durham inverted tubes [11]. Cell morphology was examined using both light microscopy and scanning electron microscopy (SEM; SU8180, Hitachi) after 3 days of growth in YM broth at 25 °C [12]. Pseudohyphae formation was assessed microscopically following incubation at 25 °C for 1 month on YM agar and corn meal agar (CMA; 2.5% corn starch and 2% agar, w/v). A cover glass was placed over the colony to create an oxygen-limited environment, facilitating the observation of pseudohyphae [13]. Growth at different temperatures was evaluated on YM agar. To investigate potential sexual stages, both strains were incubated individually and in mixed cultures on YM agar, CMA, Fowell’s acetate agar and malt extract agar (MEA; 5% malt extract and 2% agar, w/v) at 25 °C for up to 2 months, with periodic microscopic examinations [12,13].

### Molecular phylogenetic analysis

DNA extraction was conducted according to the procedure described by Kurtzman [14]. The D1/D2 domains of the LSU rRNA and the ITS region were amplified and sequenced using the primers NL1 and NL4 (for the D1/D2 domain) [15] and ITS1 and ITS4 (for the ITS region) [16], respectively. Yeast strains were primarily identified through the blast in GenBank using their D1/D2 and ITS sequences as queries [17]. Subsequently, the sequences of related type strains were retrieved and downloaded from GenBank and aligned using mega 7.0 with manual adjustment.

Phylogenetic trees were constructed using the maximum-likelihood (ML), the neighbour-joining (NJ) and the maximum-parsimony (MP) methods [18,20] in mega 7.0 based on sequences of the D1/D2 domains of LSU rRNA gene and the concatenated D1/D2-ITS region. For the ML analysis, the general time reversible model was applied, while the Kimura-2 parameter model was used for distance correction in the NJ analysis. *Cryptococcus amylolentus* CBS 6039^T^ was used as the outgroup. The robustness of the phylogenetic trees was assessed using bootstrap analysis with 1,000 replicates [21], and only bootstrap values exceeding 50% were indicated on the resulting trees [22].

## Result and discussion

### Molecular phylogenetic analyses

Sequence analysis of the D1/D2 domains of the LSU rRNA gene and the ITS region revealed that strains 21S12 and 12S11 exhibit significant divergence from all recognized yeast species. Phylogenetically, the strains were most closely related to *F. mujuensis* CBS 10308^T^, with sequence similarities of 95.73% for the D1/D2 domains and 91.59% for the ITS region. The divergence between the D1/D2 regions of the new isolates and *F. mujuensis CBS* 10308^T^ comprised 28 nt substitutions and 2 indels, while the ITS region showed 28 substitutions and 10 indels. Notably, strains 21S12 and 12S11 shared identical D1/D2 and ITS sequences, confirming that they belong to the same species. As they were isolated from distinct samples (21S12: twelfth colony from Sample 21; 12S11: eleventh colony from Sample 12), they represent non-clonal isolates. These results support the conclusion that the two strains represent a novel species within the genus *Fonsecazyma*.

**Fig. 1.**
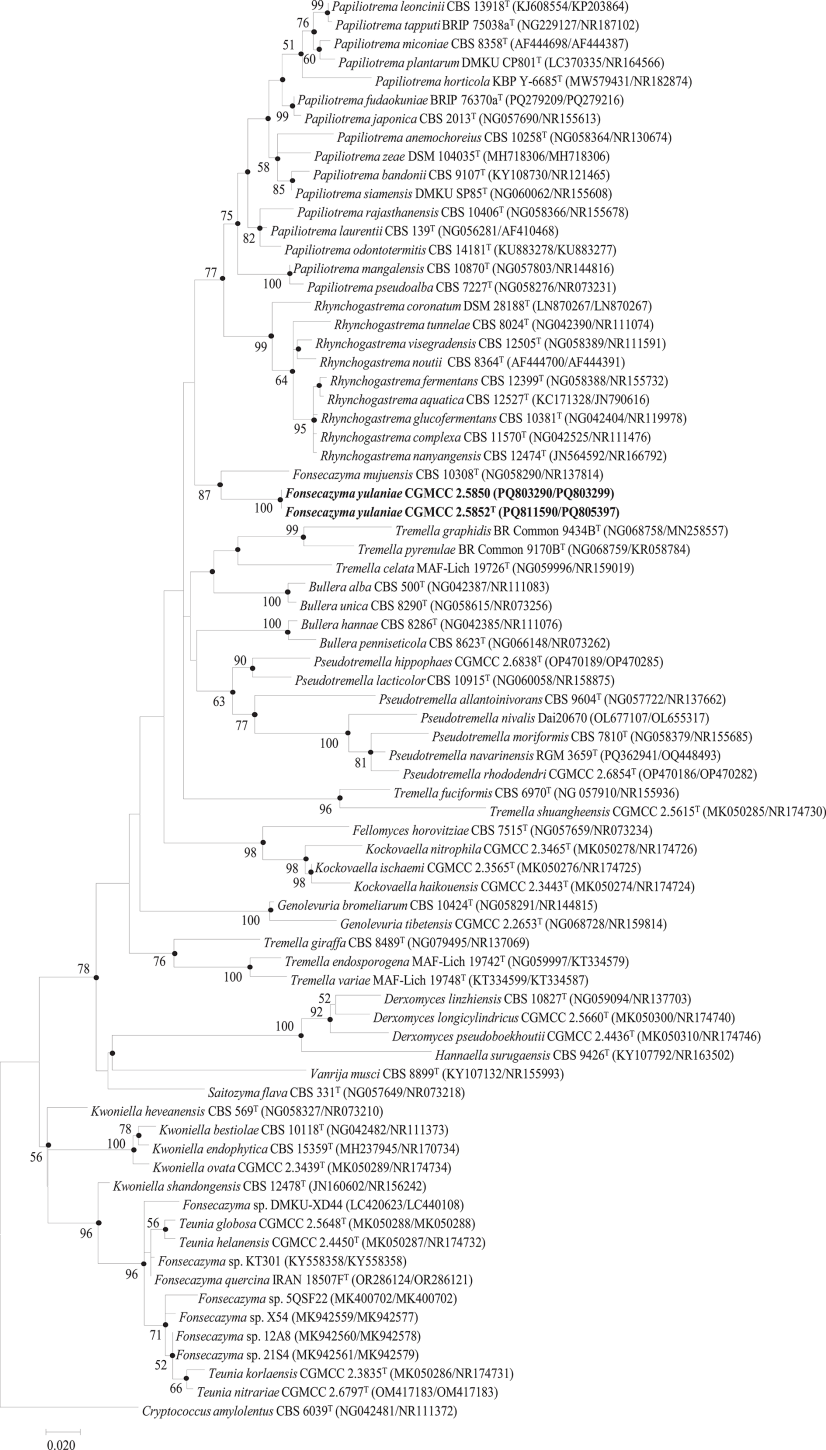
ML phylogenetic tree based on concatenated sequences of the D1/D2 domains of the LSU rRNA gene and the ITS region, showing the phylogenetic placement of *F. yulaniae* sp. nov. among related genera. Bootstrap values ≥50% (from 1,000 replicates) are indicated at the nodes. Filled circles represent nodes supported by all three methods: ML, NJ and MP. *C. amylolentus* CBS 6039^T^ was used as the outgroup. Bar, 0.02 substitutions per nt position.

The phylogenetic position of strains 21S12 and 12S11 was determined using NJ, ML and MP analyses based on D1/D2 domains of the LSU rRNA gene (Fig. S1, available in the online Supplementary Material) and the concatenated sequences of the D1/D2 domains of the LSU rRNA gene and the ITS region, respectively. As shown in Fig. 1, the ML analysis placed the two novel strains adjacent to *F. mujuensis* CBS 10308^T^, forming a well-supported clade with a bootstrap value of 87%. This phylogenetic placement was further corroborated by the NJ and MP methods, which yielded consistent topologies (Fig. S2).

Based on the significant genetic divergence from *F. mujuensis* and their distinct phylogenetic position, strains 21S12 and 12S11 are proposed to represent a novel species within the genus *Fonsecazyma*, for which the name *F. yulaniae* sp. nov. is designated.

A point worthy of note is that, despite the strain IRAN 18507F was initially described as a novel species within the genus *Fonsecazyma*, designated as *F. quercina* sp. nov., phylogenetic analysis revealed that this strain clustered within the *Teunia* clade, demonstrating closer phylogenetic affinity to members of the genus *Teunia* than to *Fonsecazyma*. This incongruence between taxonomic designation and molecular phylogeny suggests that the current classification of *F. quercina* may require re-evaluation as additional genomic data from related taxa become available.

### Phenotypic characteristics

**Fig. 2.**
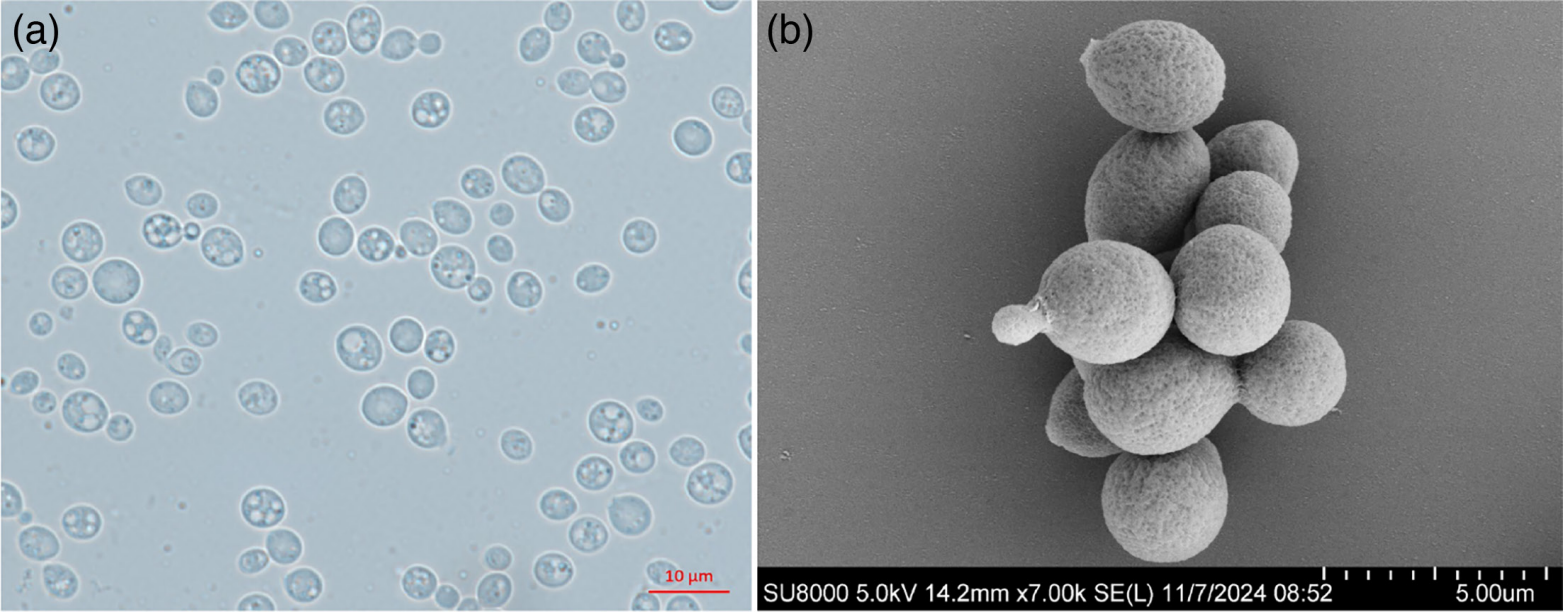
Cell morphology of *F. yulaniae* sp. nov. 21S12^T^ observed by optical microscope (**a**) and SEM (**b**) after 3 days of growth at 25 °C in YM broth.

After 3 days of growth in YM broth at 25 °C, multilateral budding was observed in the new species. When observed under SEM, the cells of strain 21S12^T^ were ellipsoidal or spherical and exhibited a rough cell surface, which may be due to the minute structures of the cell wall (Fig. 2). Physiologically, the novel species shares several traits with its closest relative, *F. mujuensis* CBS 10308^T^. Both species can assimilate d-glucose, d-ribose, d-xylose, l-arabinose, sucrose, maltose, methyl-*α*-d-glucoside, trehalose, cellobiose, raffinose, melezitose, soluble starch, inositol, succinic acid, ethylamine hydrochloride and cadaverine. Conversely, they are unable to assimilate l-sorbose, methanol, potassium nitrate or sodium nitrite, and neither species grows at 35 °C. The novel species can be distinguished from its closest relative, *F. mujuensis* CBS 10308^T^, by several key physiological and biochemical traits. Specifically, the new species demonstrates the ability to assimilate inulin, creatine (weakly) and creatinine, capabilities absent in *F. mujuensis* (Table 1). Conversely, the novel species cannot assimilate melibiose, lactose, erythritol or dl-lactate, all of which are assimilated by *F. mujuensis* (Table 1). Additionally, the novel species exhibits unique growth capabilities: it can grow in a vitamin-free medium and in a medium supplemented with 50% glucose, conditions under which *F. mujuensis* fails to grow. In contrast, *F. mujuensis* can grow in the presence of 10% NaCl, a trait not observed in the novel species.

**Table 1.** Phenotypic characteristics differentiating *F. yulaniae* sp. nov. from its closest relative, *F. mujuensis* CBS 10308^T^ Species: 1, *F. yulaniae* sp. nov. 21S12^T^; 2, *F. mujuensis* CBS 10308^T^; 3, *F. quercina* IRAN 18507 F^T^. +, Positive; –, negative; w, weakly positive; d, delayed; dw, delayed weak; n, not determined. Data for *F. mujuensis* CBS 10308^T^ and *F. quercina* IRAN 18507 F^T^ were taken from [23] and [3], respectively.

Characteristic	1	2	3
Assimilation of:			
Galactose	+	+	−
Cellobiose	+	+	w
Lactose	−	+	−
Melibiose	−	d	−
Inulin	+	−	n
d-Ribose	+	+	−
l-Arabinose	+	+	−
l-Rhamnose	+	+	w
Ethanol	+	n	n
Glycerol	w	d	n
Erythritol	−	+	n
dl-Lactate	−	dw	n
d-Glucitol	+	+	w
Salicin	w	+	n
l-Lysine	+	d	n
Creatine	w	−	n
Creatinine	+	−	n
Other tests:			
Vitamin-free	+	−	n
50% glucose	+	−	n
10% NaCl	−	+	+

### Ecology

The genus *Fonsecazyma* currently comprises only two known species, *F. mujuensis* and *F. quercina. F. mujuensis* was originally isolated from wild rabbit faeces and is hypothesized to play a role in the decomposition of organic matter in faecal environments [23]. *F. quercina* was first isolated from a Persian oak branch, and it is speculated that this species may decompose organic matter within the branches, convert it into simple compounds and supply these compounds back to the tree, thereby promoting material cycling. In contrast, the novel species, *F. yulaniae* sp. nov., was isolated from the flowers of *Y. denudata*, suggesting a distinct ecological niche and a potential association with floral ecosystems.

As for the investigation of the yeast community structure in floral environments, we collected various flower samples (including petals and floral cores) from the Beijing Olympic Forest Park, PR China, to isolate the yeast strains. The yeast strains we isolated from flower samples belonged totally to 70 species of 42 genera. The predominant yeast species inhabiting the floral niches were *Starmerella bombicola*, *Teunomyces globosus*, *Kwoniella ovata*, *Aureobasidium pullulans*, *Vishniacozyma tephrensis* and *Cystobasidium pinicola*. The detailed information of the yeast strains is listed in Table S1. The study of floricolous yeasts is an emerging field that seeks to understand the biodiversity, ecological roles and interactions of yeasts within floral environments [24]. Investigating these unique ecosystems not only expands our knowledge of yeast diversity but also provides insights into the complex relationships between micro-organisms and their habitats.

## Description of *Fonsecazyma yulaniae* Y.J. Liao, X. Zhang and A.H. Li sp. nov.

*Fonsecazyma yulaniae* (yu.la'ni.ae. N.L. gen. n. *yulaniae*, of the plant species *Y. denudata*, from which flowers it has been isolated).

The cells are ovoid to ellipsoid (2.1–5.4×2.8–5.9 µm) and occur singly or in pairs after 3 days of growth in YM broth at 25 °C. Multilateral budding is observed (Fig. 2a). After growth in YM broth for 1 month at 25 °C, a ring and a sediment are formed. The streak culture is pale yellow-cream, glistening, butyrous and smooth with an entire margin after 1 month of incubation on YM agar at 25 °C. No hyphae or pseudohyphae are observed on CMA, potato dextrose agar (PDA) and YM agar. Additionally, sexual reproduction is not observed on CMA, MEA, Fowell’s acetate agar or PDA agar under the same conditions. The strain could grow (weak) at 30 °C but could not grow at 35 °C. Growth in YM broth with 50% (w/v) glucose and in the vitamin-free medium is positive. Growth in YM broth with 10% (w/v) sodium chloride is negative. Additionally, the strain shows positive urease activity and a positive diazonium blue B reaction. Fermentation of glucose, galactose, sucrose, maltose, lactose and raffinose was absent. Glucose, galactose, sucrose, maltose, cellobiose, trehalose, raffinose, melezitose, inulin, soluble starch, d-xylose, d-ribose, l-arabinose, l-rhamnose, ethanol, glycerol (weak), ribitol, galactitol, d-mannitol, d-glucitol, methyl-*α*-d-glucoside, salicin (weak), succinic acid and inositol are assimilated as sole carbon sources. l-Sorbose, lactose, melibiose, methanol, erythritol, dl-lactate and citrate are not assimilated. Ethylamine hydrochloride, cadaverine, l-lysine, creatine (weak) and creatinine are assimilated as sole nitrogen sources, whereas nitrate and nitrite are not assimilated.

The holotype, CGMCC 2.5852^T^, was isolated from the flowers of *Y. denudata* collected from the Beijing Olympic Forest Park, PR China. It has been deposited in a metabolically inactive state in the CGMCC, Beijing, PR China. The ex-type culture has been deposited in the Biological Resource Center, NITE (NBRC), Japan, as NBRC 114206. The GenBank/EMBL/DDBJ accession number for the 26S rRNA gene D1/D2 domain and the ITS sequence of strain CGMCC 2.5852^T^ are PQ811590 and PQ805397. Its taxonomic description has been registered in Fungal Names (FN572294).

## Supplementary material

10.1099/ijsem.0.006830 Uncited Supplementary Material 1.

## Abbreviations

CGMCC: China General Microbiological Culture Collection Center
CMA: corn meal agar
ITS: internal transcribed spacer
LSU: large subunit
MEA: malt extract agar
ML: maximum-likelihood
MP: maximum-parsimony
NJ: neighbour-joining
PDA: potato dextrose agar
SEM: scanning electron microscopy
YM: yeast extract-malt extract
