# Supplementary material for: Fonsecazyma yulaniae sp. nov., a yeast species isolated from flowers
Source: Int J Syst Evol Microbiol. 2025 Jun 26;75(6):006830. doi: 10.1099/ijsem.0.006830 (PMC12282027; doi:10.1099/ijsem.0.006830)
Supplement: Uncited Supplementary Material 1. [file ijsem-75-06830-s001.pdf]

Fonsecazyma yulaniae sp. nov., a yeast species isolated from flowers

You-Jun Liao[1,2], Xuan Zhang[1,2], Zi-Xuan Liu[2], Rui Wang[3], Ya-Jing Yu[2], Lu Xue[1*], Ai-Hua Li[2*]

1. School of Biotechnology and Food Science, Tianjin University of Commerce, Tianjin, PR China
2. China General Microbiological Culture Collection Center (CGMCC), Institute of Microbiology, Chinese Academy of Sciences, Beijing 100101, PR China
3. Tianjin Institute of Industrial Biotechnology, Chinese Academy of Sciences, Tianjin 300308, PR China

Corresponding author:

* Ai-Hua Li: Email, liah@im.ac.cn; Tel, 86-10-6480-6073;

Lu Xue: E-mail, xuelu@tjcu.edu.cn

Keywords: flower; *Fonsecazyma*; novel species; phylogeny.

Author Notes: The GenBank/EMBL/DDBJ accession numbers for the sequences of the D1/D2 domain of the LSU rRNA gene and the ITS region determined in this study are PQ811590 and PQ805397 for strain 21S12^T (CGMCC 2.5852^T), and PQ803290 and PQ803299 for strain 12S11 (CGMCC 2.5850), respectively.

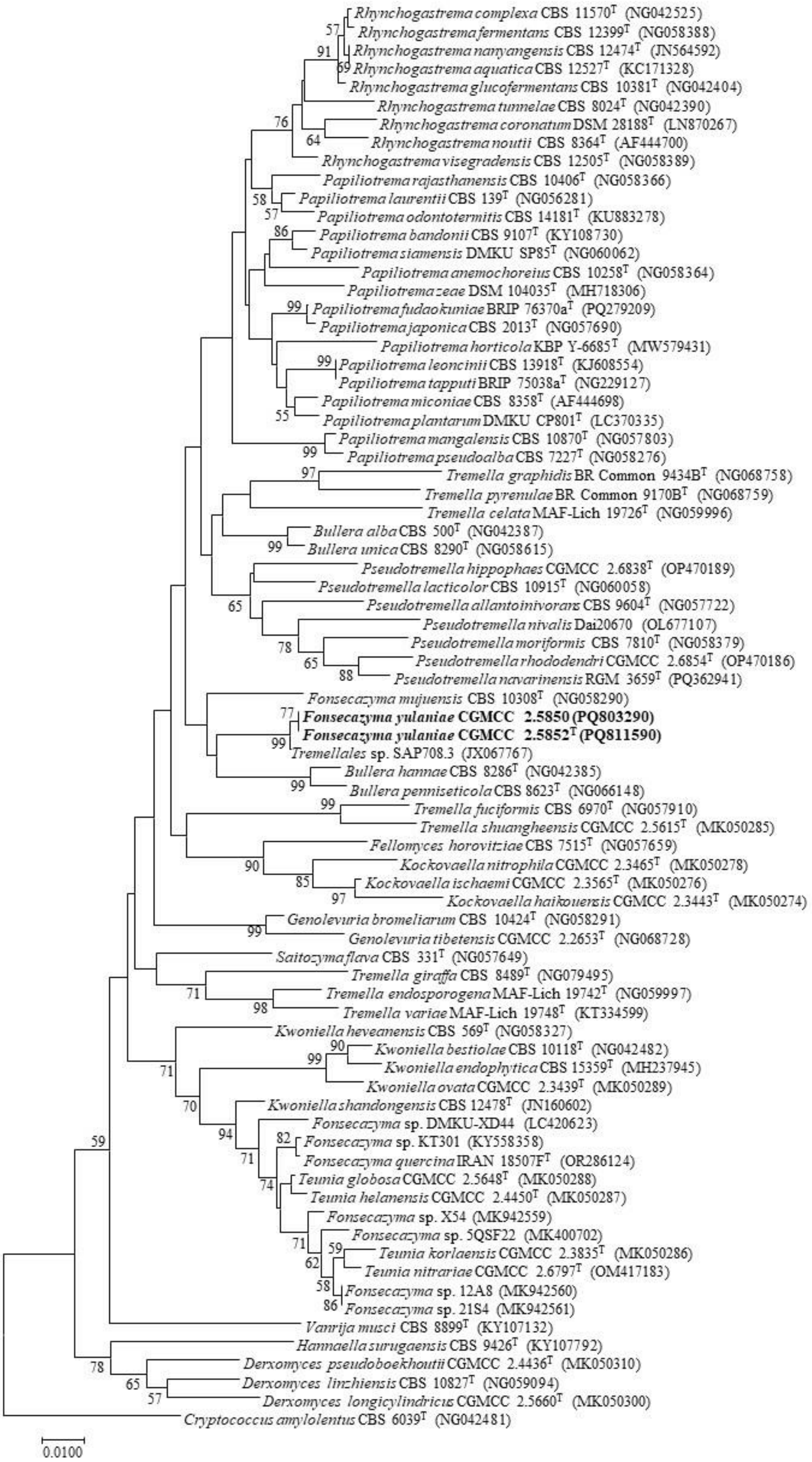

Figure S1. Neighbor-joining (NJ) phylogenetic tree based on the D1/D2 domains of the LSU rRNA gene, showing the phylogenetic placement of *Fonsecazyma yulaniae* sp. nov. among related genera. Bootstrap values ≥50% (from 1,000 replicates) are indicated at the nodes.

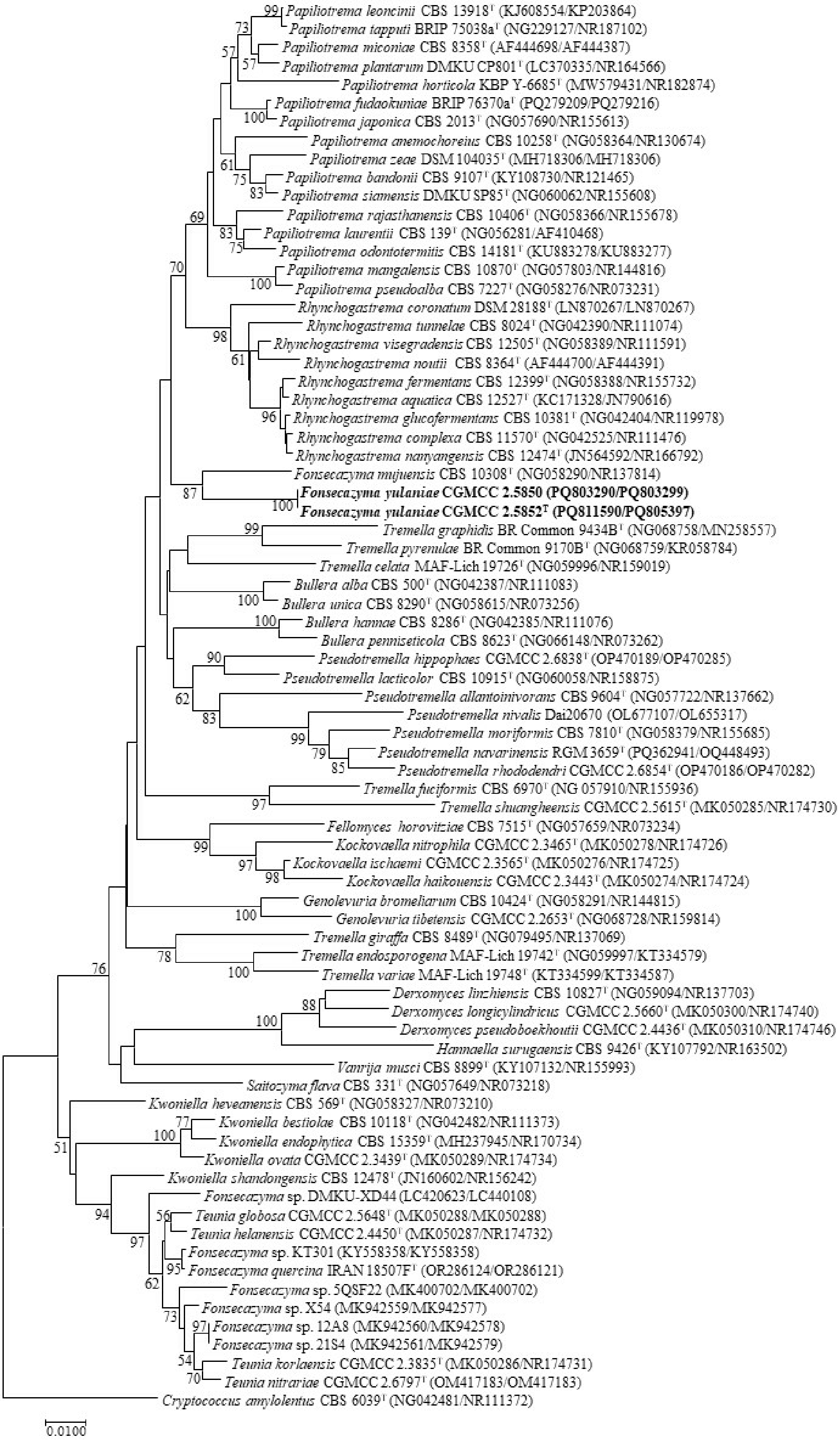

Papiliotrema leoncinii CBS 13918T (KJ608554/KP203864)
Papiliotrema tapputi BRIP 75038aT (NG229127/NR187102)
Papiliotrema miconiae CBS 8358T (AF444698/AF444387)
Papiliotrema plantarum DMKU CP801T (LC370335/NR164566)
Papiliotrema horticola KBP Y-6685T (MW579431/NR182874)
Papiliotrema fudaokuniae BRIP 76370aT (PQ279209/PQ279216)
Papiliotrema japonica CBS 2013T (NG057690/NR155613)
Papiliotrema anemochoreius CBS 10258T (NG058364/NR130674)
Papiliotrema zeae DSM 104035T (MH718306/MH718306)
Papiliotrema bandonii CBS 9107T (KY108730/NR121465)
Papiliotrema siamensis DMKU SP85T (NG060062/NR155608)
Papiliotrema rajasthanensis CBS 10406T (NG058366/NR155678)
Papiliotrema laurentii CBS 139T (NG056281/AF410468)
Papiliotrema odontotermitis CBS 14181T (KU883278/KU883277)
Papiliotrema mangalensis CBS 10870T (NG057803/NR144816)
Papiliotrema pseudoalba CBS 7227T (NG058276/NR073231)
Rhynchogastrema coronatum DSM 28188T (LN870267/LN870267)
Rhynchogastrema tunnelae CBS 8024T (NG042390/NR111074)
Rhynchogastrema visegradensis CBS 12505T (NG058389/NR111591)
Rhynchogastrema noutii CBS 8364T (AF444700/AF444391)
Rhynchogastrema fermentans CBS 12399T (NG058388/NR155732)
Rhynchogastrema aquatica CBS 12527T (KC171328/JN790616)
Rhynchogastrema glucofermentans CBS 10381T (NG042404/NR119978)
Rhynchogastrema complexa CBS 11570T (NG042525/NR111476)
Rhynchogastrema nanyangensis CBS 12474T (JN564592/NR166792)
Fonsecazyma mujuensis CBS 10308T (NG058290/NR137814)
Fonsecazyma yulaniae CGMCC 2.5850 (PQ803290/PQ803299)
Fonsecazyma yulaniae CGMCC 2.5852T (PQ811590/PQ805397)
Tremella graphidis BR Common 9434BT (NG068758/MN258557)
Tremella pyrenulae BR Common 9170BT (NG068759/KR058784)
Tremella celata MAF-Lich 19726T (NG059996/NR159019)
Bullera alba CBS 500T (NG042387/NR111083)
Bullera unica CBS 8290T (NG058615/NR073256)
Bullera hannae CBS 8286T (NG042385/NR111076)
Bullera penniseticola CBS 8623T (NG066148/NR073262)
Pseudotremella hippophaes CGMCC 2.6838T (OP470189/OP470285)
Pseudotremella lacticolor CBS 10915T (NG060058/NR158875)
Pseudotremella allantoinivorans CBS 9604T (NG057722/NR137662)
Pseudotremella nivalis Dai20670 (OL677107/OL655317)
Pseudotremella moriformis CBS 7810T (NG058379/NR155685)
Pseudotremella navarinensis RGM 3659T (PQ362941/OQ448493)
Pseudotremella rhododendri CGMCC 2.6854T (OP470186/OP470282)
Tremella fuciformis CBS 6970T (NG 057910/NR155936)
Tremella shuangheensis CGMCC 2.5615T (MK050285/NR174730)
Fellomyces horovitziae CBS 7515T (NG057659/NR073234)
Kockovaella nitrophila CGMCC 2.3465T (MK050278/NR174726)
Kockovaella ischaemi CGMCC 2.3565T (MK050276/NR174725)
Kockovaella haikouensis CGMCC 2.3443T (MK050274/NR174724)
Genolevuria bromeliarum CBS 10424T (NG058291/NR144815)
Genolevuria tibetensis CGMCC 2.2653T (NG068728/NR159814)
Tremella giraffa CBS 8489T (NG079495/NR137069)
Tremella endosporogena MAF-Lich 19742T (NG059997/KT334579)
Tremella variae MAF-Lich 19748T (KT334599/KT334587)
Derxomyces linzhiensis CBS 10827T (NG059094/NR137703)
Derxomyces longicylindricus CGMCC 2.5660T (MK050300/NR174740)
Derxomyces pseudoboekhoutii CGMCC 2.4436T (MK050310/NR174746)
Hannaella surugaensis CBS 9426T (KY107792/NR163502)
Vanrija musci CBS 8899T (KY107132/NR155993)
Saitozyma flava CBS 331T (NG057649/NR073218)
Kwoniella heveanensis CBS 569T (NG058327/NR073210)
Kwoniella bestiolae CBS 10118T (NG042482/NR111373)
Kwoniella endophytica CBS 15359T (MH237945/NR170734)
Kwoniella ovata CGMCC 2.3439T (MK050289/NR174734)
Kwoniella shandongensis CBS 12478T (JN160602/NR156242)
Fonsecazyma sp. DMKU-XD44 (LC420623/LC440108)
Teunia globosa CGMCC 2.5648T (MK050288/MK050288)
Teunia helanensis CGMCC 2.4450T (MK050287/NR174732)
Fonsecazyma sp. KT301 (KY558358/KY558358)
Fonsecazyma quercina IRAN 18507FT (OR286124/OR286121)
Fonsecazyma sp. 5QSF22 (MK400702/MK400702)
Fonsecazyma sp. X54 (MK942559/MK942577)
Fonsecazyma sp. 12A8 (MK942560/MK942578)
Fonsecazyma sp. 21S4 (MK942561/MK942579)
Teunia korlaensis CGMCC 2.3835T (MK050286/NR174731)
Teunia nitrariae CGMCC 2.6797T (OM417183/OM417183)
Cryptococcus amylolentus CBS 6039T (NG042481/NR111372)
0.0100

a

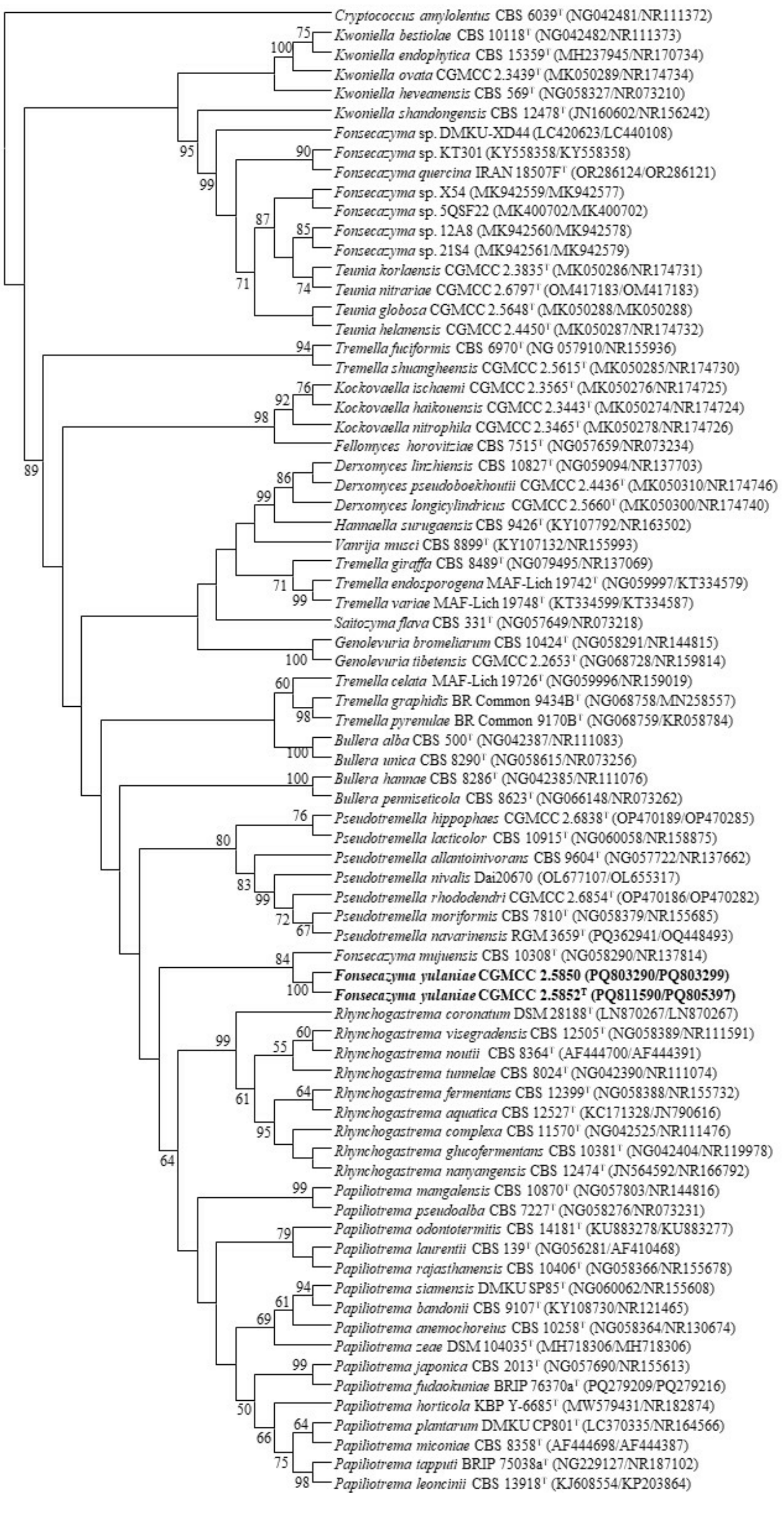

b

Figure S2. Neighbor-joining (NJ) and maximum-parsimony (MP) phylogenetic tree based on concatenated sequences of the D1/D2 domains-ITS region, showing the phylogenetic placement of *Fonsecazyma yulaniae* sp. nov. among related genera. Bootstrap values ≥50% (from 1,000 replicates) are indicated at the nodes.

Table S1. The yeast strains isolated from floral niches

**Species**	**Numbers**
Starmerella bombicola	30
Teunia globosa	29
Kwoniella ovata	27
Aureobasidium pini	22
Vishniacozyma tephrensis	16
Cystobasidium pinicola	16
Dioszegia zsoltii	12
Erythrobasidium primogenitum	10
Cystofilobasidium infirmominiatum	10
Vishniacozyma carnescens	9
Cystofilobasidium macerans	9
Aureobasidium namibiae	8
Vishniacozyma victoriae	7
